# Reducing and managing faecal incontinence in people with advanced dementia who are resident in care homes: protocol for a realist synthesis

**DOI:** 10.1136/bmjopen-2015-007728

**Published:** 2015-07-10

**Authors:** Claire Goodman, Jo Rycroft Malone, Christine Norton, Danielle Harari, Rowan Harwood, Brenda Roe, Bridget Russell, Mandy Fader, Marina Buswell, Vari M Drennan, Frances Bunn

**Affiliations:** 1 Centre for Research in Primary and Community Care, University of Hertfordshire, Hatfield, UK; 2 School of Healthcare Sciences, Bangor University School of Health Care Sciences, Bangor, UK; 3 Florence Nightingale School of Nursing & Midwifery, King's College London, London, UK; 4 Division of Health and Social Care, Department of Ageing and Health, Guys and St Thomas’, King's College London, London, UK; 5 Health Care of Older People Nottingham University Hospitals NHS Trust, University of Nottingham, Nottingham, UK; 6 Faculty of Health and Social Care, Edge Hill University of Manchester, Manchester, UK; 7 Department of Health Sciences, University of Southampton, Southampton, UK; 8 Faculty of Health, Social Care and Education, St Georges University of London and Kingston University, UK

**Keywords:** GERIATRIC MEDICINE

## Abstract

**Introduction:**

Faecal incontinence (FI) is the involuntary loss of liquid or solid stool that is a social or hygienic problem. The prevalence of FI in residents of care homes is high, but it is not an inevitable consequence of old age or dementia. There is good evidence on risk factors, but few studies provide evidence about effective interventions. There is a need to understand how, why, and in what circumstances particular programmes to reduce and manage FI are effective (or not) for people with dementia. The purpose of this review is to identify which (elements of the) interventions could potentially be effective, and examine the barriers and facilitators to the acceptability, uptake and implementation of interventions designed to address FI in people with dementia who are resident in care homes.

**Methods and analysis:**

A realist synthesis approach to review the evidence will be used which will include studies on continence, person-centred care, implementation research in care homes, workforce and research on care home culture. An iterative four-stage approach is planned. Phase 1: development of an initial programme theory or theories that will be ‘tested’ through a first scoping of the literature and consultation with five stakeholder groups (care home providers, user representatives, academics and practice educators, clinicians with a special interest in FI and continence specialists). Phase 2: a systematic search and analysis of published and unpublished evidence to test and develop the programme theories identified in phase 1. Phase 3: validation of programme theory/ies with a purposive sample of participants from phase 1.

**Ethics and dissemination:**

The overall protocol does not require ethical review. The University research ethics committee will review interviews conducted as part of phase 1 and 3. The final fourth phase will synthesise and develop recommendations for practice and develop testable hypotheses for further research.

## Introduction

In the UK, care homes are the main providers of long-term care for older people. Approximately 17 500 care homes are home to about 487 000 older people, the majority women aged 80 years or older.^1^

It is estimated that as many as 80% of residents may have dementia, though this is not always documented.^2^ For the purposes of this paper, ‘care home’ and ‘long-term care’ refers to residential care provided to older people, who require help with personal care and who are unable to be supported in their own home for reasons of frailty, lack of mental capacity and/or functional limitations. It includes settings that have on-site nursing provision and those that do not. In the UK, this care is provided by a combination of for profit and not-for-profit providers. It is a sector that is diverse, varying in size, ownership, funding sources, focus and organisational culture.

Faecal incontinence (FI) is the involuntary loss of liquid or solid stool; this is a social or personal hygiene problem.^3^ The prevalence of FI in people aged over 80 years is estimated to range from 12% to 22%.^4^ ^5^ In a cohort study of primary care patients, the rate of diagnosis of FI in people with dementia is four times that in a matched sample without a diagnosis of dementia.^6^ Dementia has also been identified as an independent risk factor for FI in several epidemiological studies.^7–9^ Estimates of the prevalence of FI and bowel-related problems in people resident in UK care homes are significantly higher than the general population. Studies in care homes suggest prevalence between 30% and 50%.^7^ ^10–14^ The level of variation is believed to reflect differences in care and how continence is defined (by frequency, amount and detection method), as well as the individual characteristics of the older people.^4^ ^5^

The current evidence about FI in care homes is mixed with some good evidence on risk factors and associations, but few intervention studies. The most recent Cochrane systematic reviews of the evidence base for FI have concluded that there are no randomised studies specifically in this patient group.^15–17^ We are interested in uncovering what interventions work for improving the care and management of FI in people with dementia who are resident in care homes, how, why and in what circumstances. There is a need to develop explanatory models for effectiveness that can draw on different sources of evidence and increase understanding about which interventions are likely to be most useful for people with dementia in care homes. By taking a realist theory-driven approach to the systematic identification, reviewing and synthesis of evidence, we aim to uncover the different underpinning mechanisms that ‘work’ on different aspects of FI (eg, amount, frequency, containment) for people with dementia who are resident in care homes. This is achieved by understanding how and why interventions and their constituent elements may impact, for whom, in which contexts and circumstances. This theory-driven understanding should be able to inform more actionable recommendations for practice and research.

## Background

National and international guidelines^18^ ^19^ emphasise that all patients with FI should be assessed for treatable causes, *regardless* of their cognitive status. Treatable causes particularly relevant to care home residents with dementia are overflow from faecal impaction, and FI from loose stools, both of which can be assessed and managed in the care home setting. For example, treating constipation has been shown to be effective in improving overflow FI, and reducing staff workload (based on soiled laundry counts) by 42% in those with effective bowel clearance.^20^ Loose stool may be due to reversible causes such as dietary intolerances, medication side-effects, including laxative use,^12^ ^21^ and antibiotic-related diarrhoea.^22^ Some patients with dementia lack cortical control of the defaecation process, tending to void formed stool following mass peristaltic movements. There is limited evidence that prompted or scheduled toileting (preferably after meals) can increase the number of continent bowel movements for care home residents.^23^ ^24^ Despite the extent of FI in care homes there is a paucity of evidence because research in continence care in care homes tends to focus on urinary incontinence.^25–28^

Problems related to FI experienced by care home residents may include dermatitis, delirium, discomfort and sometimes unplanned hospital admissions.^29^ ^30^ FI frequency is strongly linked to negative impact on quality of life.^18^ ^31–34^ It also affects opportunities for social interaction and stimulation, and can compound the isolation already created by living with dementia.^35^ Dealing with FI may also affect care home staff turnover and morale in a workforce that is already low paid^36^ with little clinical support.

In 2012, a specific care home continence audit, educational and care planning tool was piloted in the UK. This highlighted some of the process and organisational problems that can be barriers to care professionals implementing FI programmes.^37^ Ageism, lack of training, pad restrictions due to cost control and poorly integrated services were identified as likely contributors to low standards of care for FI. A review of English local continence guidelines^38^ revealed a paucity of dementia-specific information. There is, however, an extensive more general care home and dementia-specific research literature, including intervention research, on the impact of the leadership, culture of care and care home routines on residents’ health and well-being.^39^ For example, contributing factors to FI include impaired mobility, stroke and diabetes. Care home studies on nutrition and hydration,^40^ patterns of meal times,^41^ medication use^42^ ^43^ and activities of daily living, for example,^44^ ^45^ all have the potential to inform implementation of FI programmes of care.

## Review objectives

We will use a realist synthesis approach to explain the effectiveness of programmes that aim to reduce and manage FI in people with dementia in care homes, and to investigate the barriers and facilitators to implementation. Specifically we will

1. Identify which (elements of the) interventions could potentially be effective, how these work (or why these do not work), on what range of outcomes (ie, organisational, resource use and patient level of care) and for whom
2. Identify the barriers and facilitators to the acceptability, uptake and implementation of interventions designed to address FI in people with dementia who are resident in care homes
3. Establish what evidence there is on the relative feasibility and (where appropriate) cost of interventions to manage faecal incontinence.

The relationship between evidence use, care experiences, quality of life, severity of a person's dementia and overall standards within care homes are not well understood or articulated.^46^ The underlying assumption of this review is that the effectiveness of programmes to address the known problems of FI in care homes is contingent not only on specific bowel-focused interventions, but also on contextually situated decision-making.^47^

Consequently, this review encompasses evidence about the physiology of FI in ageing populations, the influence of dementia research on the management of incontinence, the relative effectiveness of different FI treatments/programmes for people with dementia, the efficacy of different types of incontinence products and the experience of living with dementia and incontinence from the perspectives of the person with dementia and their paid and unpaid carers. Interventions of interest include those that focus on assessment and recovery of physiological function,^16^ medication review,^48^ ^49^ toileting regimes,^50^ those that address system-wide issues about access to assessment and treatment^51^ as well as those that, by association, have the potential to improve bowel-related care (eg, studies on dignity, interventions to improve communication with people with advanced dementia, strength and mobility, nutrition, oral hygiene and speech and language assessment).

## Methods

Realist synthesis is a systematic, theory-driven approach designed to make sense of diverse evidence about complex interventions applied in different settings.^47^ ^52–55^ An iterative four-stage approach is proposed^56^ ^57^ and captured in the RAMESES publication standards.^58^ The assumption is that a review on programmes to manage FI has to consider complementary evidence. This includes evidence on the effectiveness (and learning from) interventions to improve continence in care homes, as well as studies that more broadly rely on healthcare professionals and care home staff working together to improve the healthcare of residents with dementia. For example, it is likely that it will be informed by theoretical work on:

- The physiological and clinical causes/associations of faecal and the consequent morbidity (eg, pressure sores, infection) in the oldest old^16^
- Theories of interprofessional learning and practice development in long-term settings, and how change in individual practice is achieved and sustained with a differentially qualified workforce^59^ ^60^
- Provision of person-centred/relationship-centred care for people with advanced dementia^61^
- Implementation theory on organisational and structural factors affecting integrated working between health and social care, and the implementation of learning and practice development in long-term care settings:^62–64^

The review optimises the knowledge and networks of the research team, and is directed by the interests of the different stakeholder groups who are represented in an advisory group. The advisory group includes representatives from care home providers (n=2) and researchers (n=2), continence specialists (n=2), dementia researchers (n=2) and representatives of care home residents and relative groups (n=2). They will advise on question relevance, emerging theory development and refinement, and the findings throughout the lifetime of the project as well as contribute to the development of recommendations and dissemination activities.

## Phase 1: defining the scope of the review—concept mining and theory development

In phase 1, the project team (CG, JRM, CN, DH, RH, BRo, BRu, MF, MB, VMD and FB), will draw on their collective expertise in continence and containment, working in care homes, dementia, frailty and interventions that support integrated working and review methodologies to work together to develop programme theories or hypotheses about why FI management programmes for people with advanced dementia living in care homes work or do not work. This phase will provide a provisional account of the impact of interventions by linking key areas of knowledge that inform how interventions are developed for this particular population.

A preliminary review will be undertaken by four members of the project team (CG, FB, MB, BRu) of a selection of key literature (eg, evaluations of relevant FI programmes, studies included in reviews) identified by the project team through key word searches and discussions with stakeholder groups, and interviews with practitioners, family carers and user representatives. Five key stakeholder groups have been identified. These are:

1. Providers of care: care home managers (up to 4 groups purposively selected to reflect range of care home provision and workforce involved in providing care),
2. Recipients of care: user representatives, for example, carer representatives and continence charities (up to 20 participants, interviewed in focus groups or individually).
3. Academics and practice educators/developers who work in care homes and/or with older people (including a focus group within a meeting of the National Care Home Research and Development Forum) (1 meeting)
4. Clinicians with a special interest in FI
5. Continence specialists, commissioners and providers of continence services (a focus group convened with representatives from the Association for Continence Advice, RCN continence Forum and the Bladder and Bowel Foundation and commissioners of continence-related resources for care homes).

The group or individual interviews will be conducted with a topic guide^65^ which will invite views as to why certain approaches to addressing FI with people with advanced dementia work, in what circumstances and why. Notes will be taken during the interviews and, with permission, digital recordings. These will be used to check the notes and aid subsequent thematic analysis.^66^ The recordings will be erased. The analysis will involve two researchers with a third to resolve discrepancies.

This will be followed by a 1 day theory building workshop in which the 11 members of the project team will meet and will begin to identify common concepts, and map and prioritise the theory identified from the searches and consultation. The findings from the initial scoping and the stakeholder interviews will be synthesised by the research team, who between themselves have expertise in dementia care, continence, technology development, care homes, implementation science and gerontological medicine. This stage will result in a theoretical/conceptual framework, and associated candidate programme theories and related contexts that will inform the remainder of the review process.

## Phase 2: retrieval, review and synthesis

For the purposes of this review FI is defined as ‘leakage of solid or liquid stool which is a social or hygienic problem’.^19^

In line with the iterative nature of a realist synthesis approach,^56^ the inclusion criteria will be refined in light of emerging data and the theoretical development in phase 1. The review is likely to include evidence sources that cover the following:

- People with dementia who have FI and are resident in a care home/long-term care.
- Studies of any intervention designed to reduce or promote recovery, reduction and management of FI (eg, improve containment, maintain skin integrity, reduce odour) and/or those that offer opportunities for transferable learning, for example, studies that focus on urinary incontinence and person-centred care interventions. Interventions may have single or multiple components, and could be delivered to individuals identified with FI or to residents identified at risk of developing FI or to staff and visiting healthcare professionals.
- Studies that provide evidence on barriers and facilitators to the implementation and uptake of interventions in care homes generally (not confined to continence), that help with understanding of programme theories and logic, or that provide evidence on underlying theories that inform the particular approach of particular interventions and the outcomes of interest; for example, studies that use codesign approaches with care home staff to introduce changes in practice.

## Search strategy

The evidence base to be reviewed and synthesised will be broad and eclectic.^52^ A diversity of evidence provides an opportunity for richer mining and greater explanation. Therefore, we will include studies of any design, including randomised controlled trials, controlled studies, effectiveness studies, uncontrolled studies, interrupted time series studies (ITS), cost-effectiveness studies, process evaluations, surveys and qualitative studies of participants’ views and experiences of interventions. We will also include unpublished and grey literature, policy documents and information about locally implemented continence programmes in the UK. Potential sources of information that will be relevant to answering the questions and aims of this review are likely to include intervention studies in care homes with people with and without advanced dementia (eg, end-of-life care, urinary incontinence), as well as transferable lessons from continence studies completed in community and hospital settings. We will, therefore, seek to maximise opportunities for identifying this literature through our consultations with different groups in phase 1 and through our project steering committee.

Our search will initially be limited from 1990 to 2014, but will include seminal papers from earlier years, such as the work of Tobin and Brocklehurst^67^ and key international papers and those identified through lateral searches. The time limit is used for several reasons. Healthcare research in care homes is a relatively recent phenomenon.^68^ Gordon *et al*^68^ identified that of 292 RCTs of interventions specifically in care homes between 1974 and 2009, half were published since 2003. Dementia research has been significantly influenced by the work of Tom Kitwood, whose seminal work was first published in 1990.^69^ Furthermore, in England and Wales, the organisation and funding of care homes was radically altered in 1993 by the implementation of the National Health Service and Community Care Act 1990. This led to progressive changes in the overall size, ownership and structure of the sector. The increased emphasis on domiciliary care has also meant that the level of dependency and frailty of older people now admitted to long-term care has increased.^70^

We will search for published and unpublished literature. All members of the project team will be involved in producing a list of relevant search terms to use in the following electronic databases:

- PubMed
- CINAHL (Cumulative Index to Nursing and Allied Health Literature),
- The Cochrane Library, including the Cochrane Database of Systematic Reviews, DARE (Database of Abstracts of Reviews of Effects), the HTA Database, NHS EED (NHS Economic Evaluation Database)
- Scopus
- SocAbs (Sociological Abstracts),
- ASSIA (Applied Social Sciences Abstract and Indexes)
- BiblioMap (The EPPI-Centre register of health promotion and public health research),
- Sirius, OpenGrey, Social Care Online, the National Research Register Archive, the National Institute of Health Research portfolio database, Google and Google Scholar.

Previous dementia reviews undertaken by members of the project team^51^ ^71–73^ have highlighted the importance of lateral searching for identifying studies for dementia-related reviews. Therefore, in addition to the above electronic database searches we will undertake the following lateral searches:

- Checking of reference lists from primary studies and relevant systematic reviews (snowballing)^74^
- Citation searches using the ‘Cited by’ option on WoS, Google Scholar and Scopus, and the ‘Related articles’ option on PubMed and WoS (‘Lateral Searching’)^75^
- Contact with experts and those with an interest in dementia, care homes and FI to uncover grey literature
- Contact with disease-specific charities and user groups, residents and relatives’ associations.

At this initial stage, we have identified three sets of search terms. One set is focused on faecal incontinence; this set was constructed from definitions used in past studies identified during our initial scoping work and on previous related systematic reviews (CN, DH, BRu). The second set of search terms is focused on care home-specific interventions developed from two reviews: on healthcare interventions in care homes, and a current realist synthesis on models of healthcare delivery to care homes (CG). The third set from systematic reviews on continence interventions for people with dementia (VMD). Search terms will be revised as the review progresses and further search terms developed as the review develops.

## Review

The guiding principle for the review is that the quality of the evidence will be judged by its contribution to the building and testing of relevant theory. The key test for the inclusion of studies is the relevance and rigour of the evidence.^52^ ^58^

The programme theories being ‘tested’ through the review are made visible through the data extraction forms.^53^ A bespoke set of data extraction forms will be developed by CG, FB, BRu and MB, and reviewed by the wider project team. These will be based on the content of the programme theory which, thereby, provides a template to interrogate the theories. If the evidence meets the test of relevance (described above), data will be extracted by one author using the form and then checked by a second member of the team. Where possible, the checking will be performed by the team member who has the most relevant expertise (eg, technical interventions to treat faecal incontinence (CN, MF), impact of care home culture (BRo, DH, JRM), uptake of innovation (JRM, VMD)). Tests of rigour are built in the bespoke data extraction tool. In addition, if appropriate and if it is felt to aid the review process, we will use critical appraisal tools appropriate to the study design; for example, checklists to assess the risk of bias in controlled studies^76^ and in qualitative studies.

Quality assessment will be undertaken by at least two reviewers (MB, BRu, CG, FB) independently with any discrepancies resolved by discussion with other members of the project team who have the relevant expertise.

## Synthesis

The analytical task is the synthesising of the extracted information from the relationships between mechanisms (eg, underlying processes, structures and entities), contexts (eg, conditions, types of setting, organisational configurations), and outcomes (ie, intended and unintended consequences and impact).^53^ Rycroft-Malone *et al*^53^ have developed an approach to synthesis by incorporating the work of^52^ Pawson^52^ and principles of realist enquiry that includes:

1. Organisation of extracted information into evidence tables representing the different bodies of literature (eg, health, long-term care, faecal incontinence, bowel care, advanced dementia)
2. Theming across the evidence tables to relate to emerging patterns (demi-regularities in realist literature) in the context, mechanism and outcomes (CMOs) seeking confirming and disconfirming evidence
3. Linking these demi-regularities (patterns) to develop hypotheses.

Data synthesis will involve individual reflection and team discussion and will

- Question the integrity of each theory
- Adjudicate between competing theories
- Consider the same theory in different settings
- Compare the stated theory with actual practice.

Coded data from the studies will then be used to confirm, refute or refine the candidate theories. Where theories fail to explain the data, alternative theories will be sought.

Once the preliminary mapping of the evidence into tables is complete, we will hold a second 1 day workshop with the whole project team. This will be carefully structured to facilitate indepth discussion of the findings, and to develop and confirm or reject the resultant hypotheses. Those confirmed will act as synthesised statements of findings around which a narrative can be developed summarising the nature of the context, mechanism and outcome links, and the characteristics of the evidence underpinning them.

## Phase 3: test and refine programme theory/ies (validation)

We will review the hypotheses and supporting evidence through interviews with two groups. This will both enhance the trustworthiness of the resultant hypotheses and also help to develop a final review narrative which will include views on the elements necessary for the effective implementation of programmes to manage FI in care homes. The two group interviews will include a minimum of 15 representatives from the five key stakeholder groups (identified in phase one above) and the 10 members of the advisory group. An interview topic guide will be developed. It will include the programme theories to date and seek views on the resonance and significance of the CMO threads, both from a practice and from a service user perspective.

## Phase 4: concluding synthesis and reporting

We will develop evidence informed framework of what is likely to work for whom and in what context in relation to programmes to manage FI for people with dementia in care homes. This will be achieved through a half-day consensus meeting.^77^ To ensure that an appropriate range of views are obtained and to allow time for discussion of the findings between representatives of different groups, we will invite up to 40 participants. This will include the study advisory group members, commissioners of continence services, clinicians, care home staff, and care home executives and user representatives. This meeting may address the following issues:

- The synergy between particular interventions and the feasibility of their implementation in care homes
- Developing and targeting different interventions with multiple impacts and outcomes for older people with dementia, NHS and care home staff and their respective organisations and policy.
- The potential of different modes of delivery.

In addition we will develop a set of actionable recommendations. The goal of the realist synthesis recommendations will be to specify the situations in which a complex intervention (ie, a FI management approach for people with dementia), modified or able to take account of certain contingencies, is likely to be able to achieve certain outcomes (eg, cure or reduction of episodes of FI or containment/management of social continence, minimisation of resident distress, appropriate use of medication, increase staff knowledge, improve residents’ quality of life, reduce FI-related pressure sores and reduce FI-related hospitalisations).

## Ethical issues

The overall protocol does not require ethical review. However, the interviews conducted as part of phase 1 and phase 3 will include family carers and service staff, and therefore will be reviewed by the University research ethics committee.

## Discussion

For older people with dementia living in care homes it is important to both address treatable causes of FI and also address effective continence care that is person and context sensitive, within a group living environment. The findings from realist synthesis of the evidence will provide a theoretical framework for practice that articulates the barriers and facilitators to effective management of FI for this population. By providing possible explanations for the way in which interventions are thought to work and how change is achieved, it will demonstrate how to tailor an intervention to the setting and patient group. We will report these in a study report for the funding body and prepare a paper for open access publication. The propositions arising from the review will also inform the design of future intervention studies, and define outstanding knowledge gaps and research needs.

## References

[1] Commission CQ. *The Adult Social Care Market and the Quality of Care Services technical report 10* London, 2010.

[2] Gordon AL, Franklin M, Bradshaw L Health status of UK care home residents: a cohort study. Age Ageing 2014;43:97–103. 10.1093/ageing/aft077 23864424 PMC3861334

[3] Norton C, Whitehead WE, Bliss DZ Management of fecal incontinence in adults. Neurourol Urodyn 2010;29:199–206. 10.1002/nau.20803 20025031

[4] Harari D Faecal incontinence in older people. Rev Clin Gerontol 2009;19:87–101. 10.1017/S0959259809990153

[5] Harari D, Husk J, Lowe D National audit of continence care: adherence to National Institute for Health and Clinical Excellence (NICE) Guidance in older versus younger adults with faecal incontinence. Age Ageing 2014;43:785–93. 10.1093/ageing/afu056 24850541

[6] Grant RL, Drennan VM, Rait G First diagnosis and management of incontinence in older people with and without dementia in primary care: a cohort study using the health improvement network primary care database. PLoS Med 2013;10:e1001505 10.1371/journal.pmed.1001505 24015113 PMC3754889

[7] Chassagne P, Landrin I, Neveu C Fecal incontinence in the institutionalized elderly: incidence, risk factors, and prognosis. Am J Med 1999;106:185–90. 10.1016/S0002-9343(98)00407-0 10230748

[8] Nakanishi N, Tatara K, Naramura H Urinary and fecal incontinence in a community-residing older population in Japan. J Am Geriatr Soc 1997;45:215–19. 10.1111/j.1532-5415.1997.tb04511.x 9033523

[9] Johanson JF, Irizarry F, Doughty A Risk factors for fecal incontinence in a nursing home population. J Clin Gastroenterol 1997;24:156–60. 10.1097/00004836-199704000-00007 9179734

[10] Borrie M, Davidson H Incontinence in institutions: costs and contributing factors. CMAJ 1992;147:322. 1643598 PMC1336202

[11] Nelson R, Furner S, Jesudason V Fecal incontinence in Wisconsin nursing homes. Dis Colon Rectum 1998;41:1226–9. 10.1007/BF02258218 9788384

[12] Brocklehurst J, Dickinson E, Windsor J Laxatives and faecal incontinence in long-term care. Nurs Stand 1999;13:32–6. 10.7748/ns1999.09.13.52.32.c2676 10693509

[13] Harrington C, Carrillo H, LaCava C Nursing facilities, staffing residents and facility deficiencies. University of California at San Francisco, Dept of Social Sciences, 1999–2005.

[14] Rodriguez NA, Sackley CM, Badger FJ Exploring the facets of continence care: a continence survey of care homes for older people in Birmingham. J Clin Nurs 2007;16:954–62. 10.1111/j.1365-2702.2006.01759.x 17462046

[15] Coggrave M, Norton C Management of faecal incontinence and constipation in adults with central neurological diseases. Cochrane Database Syst Rev 2013;12:CD002115. 10.1002/14651858.CD002115.pub4 24347087

[16] Norton C, Cody JD Biofeedback and/or sphincter exercises for the treatment of faecal incontinence in adults. Cochrane Database Syst Rev 2012;7:CD002111. 10.1002/14651858.CD002111.pub3 PMC11365095 22786479

[17] Maeda Y, Laurberg S, Norton C Perianal injectable bulking agents as treatment for faecal incontinence in adults. Cochrane Database Syst Rev 2010;5:CD007959. 10.1002/14651858.CD007959.pub2 20464759

[18] NICE. Faecal incontinence: the management of faecal incontinence in adults. 2007. 21309139

[19] Norton C, Whitehead W Conservative and pharmacological management of faecal incontinence in adults. In: Wein A, ed. Incontinence. Health Publications Ltd, 2009.

[20] Chassagne P, Jego A, Gloc P Does treatment of constipation improve faecal incontinence in institutionalized elderly patients? Age Ageing 2000;29:159–64. 10.1093/ageing/29.2.159 10791451

[21] Goodman C, Davies SL, Norton C Collaborating with primary care: promoting shared working between district nurses and care home staff. In: Froggatt, et al. (eds). Understanding care homes: a research and development perspective. London: Jessica Kingsley Publishers, 2009.

[22] Tal S, Gurevich A, Guller V Risk factors for recurrence of clostridium difficile-associated diarrhea in the elderly. Scand J Infect Dis 2002;34:594–7. 10.1080/00365540210147525 12238576

[23] Ouslander JG, Simmons S, Schnelle J Effects of prompted voiding on fecal continence among nursing home residents. J Am Geriatr Soc 1996;44:424–8. 10.1111/j.1532-5415.1996.tb06415.x 8636590

[24] Bates-Jensen BM, Alessi CA, Al-Samarrai NR The effects of an exercise and incontinence intervention on skin health outcomes in nursing home residents. J Am Geriatr Soc 2003;51:348–55. 10.1046/j.1532-5415.2003.51108.x 12588578

[25] Flanagan L, Roe B, Jack B Systematic review of care intervention studies for the management of incontinence and promotion of continence in older people in care homes with urinary incontinence as the primary focus (1966–2010). Geriatr Gerontol Int 2012;12:600–11. 10.1111/j.1447-0594.2012.00875.x 22672329

[26] Flanagan L, Roe B, Jack B Factors with the management of incontinence and promotion of continence in older people in care homes. J Adv Nurs 2014;70:476–96. 10.1111/jan.12220 23889325

[27] Roe B, Flanagan L, Jack B Systematic review of the management of incontinence and promotion of continence in older people in care homes: descriptive studies with urinary incontinence as primary focus. J Adv Nurs 2011;67:228–50. 10.1111/j.1365-2648.2010.05481.x 21105895 PMC3132440

[28] Roe B, Flanagan L, Jack B Systematic review of descriptive studies that investigated associated factors with the management of incontinence in older people in care homes. Int J Older People Nurs 2013;8:29–49. 10.1111/j.1748-3743.2011.00300.x 22182302

[29] Morley JE, Steinberg KE Diarrhea in long-term care: a messy problem. J Am Med Dir Assoc 2009;10:213–17. 10.1016/j.jamda.2009.01.007 19426933

[30] Gray M, Beeckman D, Bliss DZ Incontinence-associated dermatitis: a comprehensive review and update. J Wound Ostomy Continence Nurs 2012;39:61–74. 10.1097/WON.0b013e31823fe246 22193141

[31] Markland AD, Goode PS, Burgio KL Incidence and risk factors for fecal incontinence in black and white older adults: a population-based study. J Am Geriatr Soc 2010;58:1341–6. 10.1111/j.1532-5415.2010.02908.x 20533967 PMC3205963

[32] Perry S, Shaw C, McGrother C Prevalence of faecal incontinence in adults aged 40 years or more living in the community. Gut 2002;50:480–4. 10.1136/gut.50.4.480 11889066 PMC1773171

[33] Bharucha AE, Zinsmeister AR, Locke GR Prevalence and burden of fecal incontinence: a population-based study in women. Gastroenterology 2005;129:42–9. 10.1053/j.gastro.2005.04.006 16012933

[34] Bharucha AE, Zinsmeister AR, Locke GR Risk factors for fecal incontinence: a population-based study in women. Am J Gastroenterol 2006;101:1305–12. 10.1111/j.1572-0241.2006.00553.x 16771954

[35] Drennan VM, Cole L, Iliffe S A taboo within a stigma? A qualitative study of managing incontinence with people with dementia living at home. BMC Geriatr 2011;11:75 10.1186/1471-2318-11-75 22081876 PMC3250935

[36] HUSSEIN S, MANTHORPE J Structural marginalisation among the long-term care workforce in England: evidence from mixed-effect models of national pay data. Ageing Soc 2014;34:21–41. 10.1017/S0144686X12000785

[37] RCP. National Audit of Continence Care (NACC). London: RCP, 2010.

[38] Drennan VM, Norrie C, Cole L Addressing incontinence for people with dementia living at home: a documentary analysis of local English community nursing service continence policies and clinical guidance. J Clin Nurs 2013;22:339–46. 10.1111/j.1365-2702.2012.04125.x 22788705

[39] Froggatt K, Davies S, Meyer J Understanding care homes: a research and development perspective. Jessica Kingsley Pub, 2009.

[40] Kenkmann A, Price GM, Bolton J Health, wellbeing and nutritional status of older people living in UK care homes: an exploratory evaluation of changes in food and drink provision. BMC Geriatr 2010;10:28 10.1186/1471-2318-10-28 20507560 PMC2890011

[41] Abbott RA, Whear R, Thompson-Coon J Effectiveness of mealtime interventions on nutritional outcomes for the elderly living in residential care: a systematic review and meta-analysis. Ageing Res Rev 2013;12:967–81. 10.1016/j.arr.2013.06.002 23811415

[42] Barber N, Alldred D, Raynor D Care homes’ use of medicines study: prevalence, causes and potential harm of medication errors in care homes for older people. Qual Saf Health Care 2009;18:341–6. 10.1136/qshc.2009.034231 19812095 PMC2762085

[43] Parsons C, Johnston S, Mathie E Potentially inappropriate prescribing in older people with dementia in care homes: a retrospective analysis. Drugs Aging 2012;29:143–55. 10.2165/11598560-000000000-00000 22204669

[44] Sackley C, Wade DT, Mant D Cluster randomized pilot controlled trial of an occupational therapy intervention for residents with stroke in UK care homes. Stroke 2006;37:2336–41. 10.1161/01.STR.0000237124.20596.92 16888263

[45] Sackley CM, Van den Berg ME, Lett K Effects of a physiotherapy and occupational therapy intervention on mobility and activity in care home residents: a cluster randomised controlled trial. BMJ 2009;339:b3123 10.1136/bmj.b3123 19723707 PMC2736373

[46] Tolson D, Rolland Y, Andrieu S International Association of Gerontology and Geriatrics: a global agenda for clinical research and quality of care in nursing homes. J Am Med Dir Assoc 2011;12:184–9. 10.1016/j.jamda.2010.12.013 21333919

[47] Rycroft-Malone J, Fontenla M, Bick D Protocol-based care: impact on roles and service delivery*. J Eval Clin Pract 2008;14:867–73. 10.1111/j.1365-2753.2008.01015.x 19018920

[48] Gage H, Goodman C, Davies SL Laxative use in care homes. J Adv Nurs 2010;66:1266–72. 10.1111/j.1365-2648.2010.05297.x 20546360

[49] Parsons C, Haydock J, Mathie E Sedative load of medications prescribed for older people with dementia in care homes. BMC Geriatr 2011;11:56 10.1186/1471-2318-11-56 21958366 PMC3197480

[50] Schnelle JF, Kapur K, Alessi C Does an exercise and incontinence intervention save healthcare costs in a nursing home population? J Am Geriatr Soc 2003;51:161–8. 10.1046/j.1532-5415.2003.51053.x 12558711

[51] Goodman C, Davies S, Dickinson A A study to develop integrated working between primary health care services and care homes final report. NIHR Service Delivery and Organisation Programme, 2013.

[52] Pawson R Evidence-based policy: a realist perspective. London: SAGE, 2006.

[53] Rycroft-Malone J, McCormack B, Hutchinson AM Realist synthesis: illustrating the method for implementation research. Implement Sci 2012;7:33 10.1186/1748-5908-7-33 22515663 PMC3514310

[54] Wong G, Greenhalgh T, Westhorp G RAMESES publication standards: realist syntheses. J Adv Nurs 2013;69:1005–22. 10.1111/jan.12095 23356726

[55] Hardwick R, Pearson M, Byng R The effectiveness and cost-effectiveness of shared care: protocol for a realist review. Syst Rev 2013;2:12 10.1186/2046-4053-2-12 23402391 PMC3599640

[56] Pawson R, Greenhalgh T, Harvey G Realist synthesis: an introduction. ESRC Research Methods Programme. Manchester: University of Manchester, 2004.

[57] Rycroft-Malone J, Burton C, Hall B Improving skills and care standards in the support workforce for older people: a realist review. BMJ Open 2014;4:e005356 10.1136/bmjopen-2014-005356 PMC4039845 24879830

[58] Wong G, Greenhalgh T, Westhorp G RAMESES publication standards: realist syntheses. BMC Med 2013;11:21 10.1186/1741-7015-11-21 23360677 PMC3558331

[59] McCormack B, Wright J, Dewar B A realist synthesis of evidence relating to practice development: findings from the literature analysis. Pract Dev Health Care 2007;6:25–55. 10.1002/pdh.211

[60] Reeves S, Tassone M, Parker K Interprofessional education: an overview of key developments in the past three decades. Work 2012;41:233–45. 22398491 10.3233/WOR-2012-1298

[61] Hughes JC, Bamford C, May C Types of centredness in health care: themes and concepts. Med Health Care Philos 2008;11:455–63. 10.1007/s11019-008-9131-5 18398697

[62] Kodner DL, Spreeuwenberg C Integrated care: meaning, logic, applications, and implications–a discussion paper. Int J Integr Care 2002;2:e12. 16896389 10.5334/ijic.67 PMC1480401

[63] Grol R, Grimshaw J From best evidence to best practice: effective implementation of change in patients’ care. Lancet 2003;362:1225–30. 10.1016/S0140-6736(03)14546-1 14568747

[64] Kitson AL, Rycroft-Malone J, Harvey G Evaluating the successful implementation of evidence into practice using the PARiHS framework: theoretical and practical challenges. Implement Sci 2008;3:1 10.1186/1748-5908-3-1 18179688 PMC2235887

[65] Bryman A Social research methods. Oxford university press, 2012.

[66] Boyatzis RE Transforming qualitative information: thematic analysis and code development. Thousand Oaks, CA; London: Sage Publications, 1998.

[67] TOBIN GW, Brocklehurst J Faecal incontinence in residential homes for the elderly: prevalence, aetiology and management. Age Ageing 1986;15:41–6. 10.1093/ageing/15.1.41 3953330

[68] Gordon AL, Logan PA, Jones RG A systematic mapping review of randomized controlled trials (RCTs) in care homes. BMC Geriatr 2012;12:31 10.1186/1471-2318-12-31 22731652 PMC3503550

[69] Kitwood T The dialectics of dementia: with particular reference to Alzheimer's disease. Ageing Soc 1990;10:177–96. 10.1017/S0144686X00008060

[70] Andrews GJ, Phillips DR Changing local geographies of private residential care for older people 1983–1999: lessons for social policy in England and Wales. Soc Sci Med 2002;55:63–78. 10.1016/S0277-9536(01)00207-6 12137189

[71] Bunn F, Goodman C, Sworn K Psychosocial factors that shape patient and carer experiences of dementia diagnosis and treatment: a systematic review of qualitative studies. PLoS Med 2012;9:e1001331 10.1371/journal.pmed.1001331 23118618 PMC3484131

[72] Trivedi D, Goodman C, Gage H The effectiveness of inter-professional working for older people living in the community: a systematic review. Health Soc Care Community 2013;21: 113–28. 10.1111/j.1365-2524.2012.01067.x 22891915

[73] Drennan VM, Greenwood N, Cole L Conservative interventions for incontinence in people with dementia or cognitive impairment, living at home: a systematic review. BMC Geriatr 2012;12:77 10.1186/1471-2318-12-77 23272951 PMC3562513

[74] Dixon-Woods M, Cavers D, Agarwal S Conducting a critical interpretive synthesis of the literature on access to healthcare by vulnerable groups. BMC Med Res Methodol 2006;6:35 10.1186/1471-2288-6-35 16872487 PMC1559637

[75] Greenhalgh T, Peacock R Effectiveness and efficiency of search methods in systematic reviews of complex evidence: audit of primary sources. BMJ 2005;331:1064–5. 10.1136/bmj.38636.593461.68 16230312 PMC1283190

[76] Higgins J, Altman DG, Gøtzsche PC The Cochrane Collaboration's tool for assessing risk of bias in randomised trials. BMJ 2011;343: d5928 10.1136/bmj.d5928 22008217 PMC3196245

[77] Jones J, Hunter D Qualitative research: consensus methods for medical and health services research. BMJ 1995;311:376–80. 10.1136/bmj.311.7001.376 7640549 PMC2550437

